# Influence of Generational Cohorts on the Preferences for Information and Communication Technologies in Latin American Patients with Obstructive Lung Diseases

**DOI:** 10.1155/2020/2489890

**Published:** 2020-01-23

**Authors:** Ivan Cherrez-Ojeda, Valeria L. Mata, Emanuel Vanegas, Miguel Felix, Jonathan A. Bernstein, Fanny M. Jiménez, Juan Carlos Calderon, Peter Chedraui, Antonio WD Gavilanes

**Affiliations:** ^1^Universidad Espíritu Santo. Samborondón, Ecuador; ^2^Respiralab Research Group, Guayaquil, Ecuador; ^3^University of Cincinnati College of Medicine, Department of Internal Medicine, Division of Immunology/Allergy Section, Cincinnati, OH, USA; ^4^Instituto de Investigación e Innovación en Salud Integral, Facultad de Ciencias Médicas, Universidad Católica de Santiago de Guayaquil, Guayaquil, Ecuador; ^5^Facultad de Ciencias de la Salud, Universidad Católica “Nuestra Señora de la Asunción”, Asunción, Paraguay; ^6^Department of Pediatrics, Maastricht University Medical Center, Maastricht, Netherlands; ^7^School of Oncology and Developmental Biology, Maastricht University, Maastricht, Netherlands

## Abstract

**Background:**

Advances in information and communication technologies (ICTs) represent a growing platform for the expansion of healthcare related services, but there is little information on how generational differences might account for distinct patterns of use and interest for ICTs. Our study aims to achieve a better understanding on how generational cohorts might influence the use and preferences for ICTs among patients with obstructive lung diseases in Latin America.

**Materials and Methods:**

We conducted an anonymous cross-sectional survey-based study, involving 968 patients with obstructive lung diseases (OLD) in Latin America. Patients rated their frequency of use and preferences of ICTs through a modified version of the Michigan Questionnaire. Chi-square test for association and adjusted regression analyses were performed.

**Results:**

Of all, 63.6% of participants had Internet access. Younger generations, in particular Generation Z and Millennials, had the highest rate of Internet access and smartphone ownership, as well as of overall frequency of ICT use. Web-based Internet was found to be the main source to seek information about the disease (36.9%) across all generational cohorts. Generation Z and Millennials presented the highest odds to be interested in using Twitter (OR 31.79 and 8.86) for receiving health-related information, and email (OR 4.87 and 4.86) as the preferred way to ask physicians information related to their disease through ICTs.

**Conclusion:**

Generational cohorts influence the use and preferences for ICTs among patients with obstructive lung diseases. Younger generational cohorts were associated with higher access to the Internet and smartphone ownership, as well as higher interest for using ICTs to receive and ask for health-related information.

## 1. Background

Nowadays, noncommunicable diseases are considered the leading cause of death in the world population, of which nearly 80% occur in low- and middle-income countries [1]. Obstructive lung diseases, particularly asthma and chronic obstructive pulmonary disease (COPD), represent a significant proportion of patients suffering from chronic conditions [2]. In response to the increasing burden of chronic diseases, healthcare systems have begun incorporating information and communication technologies (ICTs), in order to improve the quality of services provided to patients, particularly in developing countries [3–5]. These ICTs are broadly defined as digital technologies that support the capture, processing, storage, and exchange of information [6]. Their use in healthcare has grown significantly due to many potential benefits, such as lower costs, better accessibility, and wider availability [3, 7].

In this regard, studies have found that more than half of patients suffering from chronic diseases have access to the Internet; additionally it is estimated that up to 72 percent of patients search for online information before or after medical appointments, meaning that patients no longer rely solely on their physicians in order to obtain health-related information [8–10]. Unfortunately, cognitive and affective barriers, such as low computer literacy, can preclude certain patients from successfully using the internet to learn about available treatments and adaptive coping skills [11, 12].The key to understanding why different groups of patients use and perceive health information from electronic sources in different ways might be related to generational cohorts [13].

Generational cohorts represent a group of individuals who were born during the same period and were influenced by specific external events that shaped similar characteristics and core values among them [14, 15]. Along with some highly historical and social events, such as World War II, the great depression, the Vietnam War, and the Fall of Berlin Wall, the appearance of the “digital age” marks a significant difference among those individuals that belong to the Z (age <24 years) and Y Generations (aged 2438 years) in comparison to those older, such as Generation X (aged 3953 years), Baby boomers (aged 5472 years), and the silent generation (aged 73–94 years) [14]. For instance, although many studies have shown that older generations are increasingly using ICTs, they continue to perceive technological advances such as the digitalization of medical records as surprising or futuristic, whereas in younger individuals it is already an expectation [13, 16].

With this in mind, our study aims to achieve a better understanding of how generational cohorts may influence the use and preferences for ICTs among patients with obstructive lung diseases. We hypothesize these generational differences might account for distinct patterns of use, as well as different levels of interest for utilizing ICTs as a tool to communicate with healthcare providers and receive health-related information.

## 2. Materials and Methods

### 2.1. Study Design

We conducted an anonymous cross-sectional survey study involving 968 patients diagnosed with either asthma or COPD in Latin American countries (Ecuador, Argentina, Mexico, Venezuela, Peru), where each of them rated themselves based on questions assessing the frequency and preferences of using ICTs. Patients demographics, such as gender race and ethnicity, as well as patterns and preferences of use of ICTs were reported. Patients were selected from public and private healthcare centers through convenience sampling, and to be included in the study they had to be diagnosed with asthma, COPD or both, and be ≥12 years old. For patients <18 years of age, a parent or legal guardian had to sign a consent, which was further revised and approved by a hospital official. Patients with psychiatric diseases, language impairment or who found it difficult to visualize the survey were excluded. Before answering our questionnaire, patients were informed about the purpose of the study and their role. During the survey, patients completed their questionnaires either by themselves or with the help of a previously trained health care provider (e.g., physician, nurse, or intern).

### 2.2. Procedures

To assess the patterns and preferences of ICT use, a Spanish version of the Michigan questionnaire was used, which was adapted for asthma and COPD patients [17]. The survey included 19 questions in total and took roughly 9 minutes to complete. Patients were asked to quantify their use for each ICT (text messaging, Facebook, Twitter, YouTube, Email, the Internet, LinkedIn and Skype) under a scale assessing frequency (daily, at least once a week, at least once a month, less than once a month, never). Furthermore, patients were asked if they used ICTs (the Internet, Facebook, Twitter, YouTube and Email) to obtain information about their disease in a dichotomic fashion (yes/no). Then, patients rated their level of interest to receive information and ask physicians about their disease through ICTs (SMS, Facebook, Twitter, YouTube and Email) under a designated scale (high, some, low or no interest). We also included a separate question which doesnot belong to the original Michigan questionnaire, to assess the interest, in a dichotomic manner (yes/no), towards asking and receiving information through WhatsApp. This question was included due to WhatsApp's high penetration in Latin America [18].

### 2.3. Ethical Considerations

This study was approved by the Ethics Committee Comité de ética e Investigación en Seres Humanos (CEISH). We obtained informed consent before participation in the survey. We guaranteed that the identity of the patient would not be revealed.

### 2.4. Statistical Analysis

For each ICT type, responses assessing frequency of use were dichotomized into “at least once a week” and “less than once a week.” Age groups were categorized in generational cohorts as follows: G.I. generation (>94 years old), silent generation (7394 years old), Baby boomers (54–72 years old), Generation X (39–53 years old), Generation Y/Millennials (24–38 years old and Generation Z (<24 years old) [13–16].

We performed a chi-squared test to evaluate the statistical significance of the associations between generational cohorts and Internet access or possession of either cell phone or smartphone. We employed the same test to evaluate the statistical significance of the associations between the generational cohorts and the frequency of use for each ICT type, the use of each ICT to obtain information about asthma and/or COPD, and the degree of interest (dichotomized into: “high or some interest” and “little or no interest”) in receiving information or asking physicians about their disease through each technology.

An adjusted logistic regression analysis was performed between the generational cohorts and the interest in receiving information and asking physicians about their disease through each ICT. Analyses were adjusted for gender, education level and years since diagnosis. The reference category for generational cohort was silent generation.

All the data were analyzed using IBM SPSS, version 24.0 software (SPSS Inc., Chicago, IL, USA). A Fisher's exact test was performed whenever there were cells with expected frequencies of less than 5. A *p*-value of less than 0.05 was considered statistically significant.

## 3. Results

Of the 968 patients enrolled in the study, 58.1% were female (Table 1). The average age was 51.1 years old (SD 21.2) with an average time of being diagnosed with either asthma or COPD of 12.8 years (Table 2). Among all the groups, Baby boomers accounted for the largest generational cohort (33.6%) (Table 1).

### 3.1. Internet Access, Owning Cell Phone, or Smartphone

In addition, 63.6% reported having access to the Internet (Supplemental Appendix, Table S1). Generation Z and Millennials reported the highest Internet access (95.1% and 93.7%, respectively). Among all groups, Millennials reported the highest percentage of owning cellphones and smartphones (96.3% and 77.5%, respectively).

### 3.2. Uses of ICT to Obtain Information about the Disease

**Figure 1:**
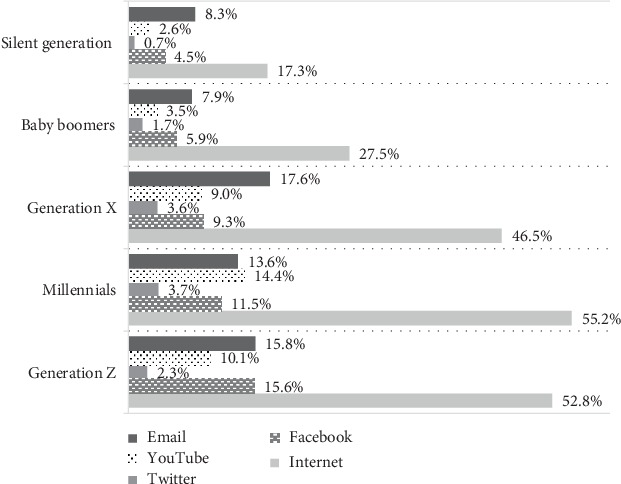
Frequency of use of ICTs to obtain health-related information by generational cohorts.

In general, SMS (63.2%), followed by the Internet (50.5%) and Facebook (44.6%) were the most frequently used ICTs (Supplemental Appendix, Table S1). Of these, the Internet was reported to have the greatest usage rate as a technology applied to seek information about their disease (36.9%) (Supplemental Appendix, Table S1). Millennials, followed by Generation Z, represented the groups with the highest rate of Internet use, either for daily activities (72.5% and 68.8%, respectively) or to obtain health information (55.2% and 52.8%, accordingly). Overall, Millennials and Generation Z had higher rates of use for every ICT (Figure 1).

### 3.3. Interest in Receiving Information and Asking Physicians about the Disease Through ICTs

SMS, followed by WhatsApp, represented the ICTs with the highest interest for receiving information about the disease (54.4% and 51.8%, respectively) and communicating with physicians (68.5% and 52.0%, respectively) (Supplemental Appendix, Table S1).

Compared to the silent generation, Generation Z presented the highest odds to be interested in receiving information and asking physicians about disease through Facebook (OR, 4.05 and 2.34), Twitter (OR, 31.79 and 3.31) and WhatsApp (OR, 6.78 and 4.60) than any other generational cohort (Table 3). In addition, Generation Z (OR, 4.87) and Millennials (OR, 4.86) were more likely to be interested in asking physicians about disease through E-mail compared to the silent generation. With respect to older cohorts, when compared to the reference category, the silent generation, Generation X (OR, 1.74) and Baby boomers (OR, 1.48) were more likely than any other group to be interested in receiving information about their disease through SMS (Table 3).

## 4. Discussion

Advances in communication technologies through the Internet and social media represent a growing platform for the expansion of healthcare related services, opening new possibilities to the increasing burden of chronically ill patients worldwide for obtaining medical information and communicating with healthcare providers [19]. The present study explored how age, analyzed through generational cohorts, influenced the use and preferences for ICTs among patients living with obstructive lung diseases in Latin America.

Certainly, the use of Internet during childhood appears to influence how each generation integrates technology in their lives [20]. Generation Z in particular, has had greater exposure during schooling years to the Internet and electronic devices than any other generation [21]. Similarly, we found that Generation Z and Millennials had the highest rate of Internet access among all generations, followed by a progressive decline as patient's age increased. The latter finding has been previously reported regarding chronic diseases in older patients who were found to use less ICTs and have lower reported Internet access compared to younger cohorts [22–24].

Among the potential benefits of the increasing use of ICTs in healthcare, the instantaneous access and wide availability of information is one that has revolutionized how patients obtain information about their medical conditions [25]. In this regard, previous studies have found an increase in the number of patients that searched the Internet for health-related information before medical appointments [25, 26]. Interestingly, this finding appears to hold true particularly among chronically ill patients, due to higher uncertainty regarding their illness, treatments and outcomes [25, 26]. In our study, we found that web-based Internet remains the main source for obtaining health-related information among patients with obstructive lung diseases across all generational cohorts, which compares to a previous study among patients with type 2 diabetes [27].

When discussing the use of specific ICTs for obtaining information among generational cohorts, we found that younger cohorts (generation Z and Millennials) had the highest overall usage of most ICTs when compared to older cohorts (Baby boomers and silent generation). Moreover, we found a similar proportion of usage for platforms such as Facebook and YouTube among generation Z and Millennials for obtaining health-related information. Based on these findings, it is important that health care providers direct patients, particularly those form younger generations, to online health information sites that are scientifically validated in order to prevent the wide dissemination of often inappropriate, dangerous or misleading content [10, 28].

Our results also indicate that younger generations showed a higher interest in receiving information and talking with physicians using most of the ICTs analyzed. For example, Generation Z and Millennials were more likely to be interested in using Twitter for receiving health-related information, and email as the preferred way to ask physicians about information related to their disease. The increasing use, wide availability, and free access to use ICTs such as Facebook and Twitter, currently considered among the most used social networks, can serve as a potential platform for health interventions [29, 30]. In fact, many groups have been created on these platforms with the purpose of assisting patients and their relatives on getting recommendations about their disease management while sharing their personal experiences with others suffering from similar conditions [31].

In contrast, we found low rates of usage for most ICTs analyzed among older generation cohorts, with the exception being SMS. The latter finding might be related to the observation that despite increasing use for certain ICTs among older generations, they continue to rely on more established resources such as text messaging [32, 33].

Finally, in the case of WhatsApp, this ICT has evolved from an instant messaging application to a platform that offers a simple, low cost and secure service, that includes sharing images, videos, files, voice messages, and even performing audio and/or video calls [34, 35]. Thus, in some countries it has been used in healthcare for teleconsultation, communication between physicians and even patient-physician interactions [7]. For instance, a Brazilian study found that almost two thirds of the participating physicians used WhatsApp to communicate with their patients [36]. In our study, we found that among all generations, Generation Z patients were more likely to be interested in both receiving information and asking physicians about their disease through WhatsApp (OR 6.78 and 4.60 respectively). This interesting finding agrees with current literature reports in which younger generational cohorts have a higher and longer usage of WhatsApp [37, 38]. However, despite the potential benefits of this ICT, several obstacles should be noted including the potential misunderstandings due to typos or interpretation errors, possible medical-legal claims without adequate insurance coverage, and lack of reimbursement policies, which could compromise and complicate the patient-doctor relationship [7].

This study has some limitations. First, it is not completely generalizable as it was not conducted in all Latin American countries, and the preferred use of ICTs in other countries could differ from those found in our study. Secondly, our results are subject to being influenced by other demographic and clinical variables such as socioeconomic status, disease severity, type of treatment, selected type of medication and others that have not been considered in the analyzes. Finally, our survey has not been validated and therefore the results could lead to biased or inaccurate conclusions. However, one strength of this study is that it encompasses a reasonably large sample size *n* = 968 of patients suffering from chronic respiratory diseases. In addition, to the best of our knowledge, this is the first study to explore the use and preferences of ICTs in Latin American among patients with obstructive lung diseases by generational cohorts.

## 5. Conclusions

Information and communication technologies represent a growing platform for the expansion of healthcare related services. They are opening up new communication channels between chronically ill patients and their physicians which are useful for dissemination of relevant medical information and treatment recommendations. In our study, web-based Internet remains the main source for obtaining health-related information among patients with obstructive lung diseases across all generational cohorts. Additionally, we found that younger generational cohorts had the highest rates of Internet access and use for most of the ICTs analyzed, followed by a progressive decline with increasing age. In particular, Generation Z and Millennials reported the highest interest for using platforms such as Twitter and Email for receiving as well as asking for health-related information, while older generations continue to rely on traditional channels of communication such as SMS.

## Figures and Tables

**Table 1:** Demographic characteristics of the surveyed population.

Characteristics	Patients (*n* = 968)*n*(%)
*Generation cohorts*	
Generation Z	114 (11.8)
Millennials	163 (16.8)
Generation X	166 (17.1)
Baby boomers	325 (33.6)
Silent generation	195 (20.1)
G.I. generation	5 (0.5)

*Gender*	
Male	405 (41.8)
Female	562 (58.1)

*Education level*	
No education	24 (2.5)
Primary school	244 (25.2)
Secondary school	383 (39.6)
Undergraduate	261 (27.0)
Postgraduate	55 (5.7)

Notes: Generation Z (<24 years old); Generation Y/Millennials (24–38 years old); Generation X (39–53 years old); Baby boomers (54–72 years old); silent generation (73–94 years old); and G.I. generation (>94 years old) [13–16].

**Table 2:** Mean age and years with the disease of surveyed population.

Characteristics	Mean (SD)

*Age (years)*	51.1 (21.2)
Generation Z	15.7 (2.5)
Millennials	28.7 (4.5)
Generation X	44.5 (4.1)
Baby boomers	61.8 (5.1)
Silent generation	77.4 (5.3)
G.I. generation	95.0 (0.7)
*Years with disease*	12.83 (13.9)

Notes: SD, standard deviation; Generation Z (<24 years old); Generation Y/Millennials (24–38 years old); Generation X (39–53 years old); Baby boomers (54–72 years old); silent generation (73–94 years old); and G.I. generation (>94 years old) [13–16].

**Table 3:** Interest in receiving and asking for information about asthma/COPD through ICTs by generational cohorts.

Variable^a^	Interest in receiving information through ICT type OR (95% CI)	Interest in asking physicians through ICT type OR (95% CI)
*SMS *(*n* = 874)		
Baby boomers	**1.48 (0.98–2.22)**	0.60 (0.35–1.02)
Generation X	**1.74 (1.06–2.88)**	**0.39 (0.23–0.66)**
Millenials	1.30 (0.78–2.15)	**0.28 (0.16–0.49)**
*Facebook *(*n* = 856)		
Generation Z	**4.05 (2.07–7.93)**	**2.34 (1.30–4.20)**
Millenials	**3.98 (2.12–7.48)**	1.71 (0.98–2.98)
Generation X	**2.30 (1.21-4.34)**	1.10 (0.62–1.93)
*Twitter *(*n* = 684)		
Generation Z	**31.79 (6.78–148.99)**	**3.31 (1.36–8.07)**
Millenials	**8.86 (1.94–40.55)**	2.00 (0.86–4.66)
*Email *(*n* = 845)		
Generation Z	1.51 (0.82–2.75)	**4.87 (2.40–9.89)**
Millenials	1.60 (0.92–2.78)	**4.86 (2.53–9.36)**
Generation X	1.63 (0.94–2.83)	**4.08 (2.13–7.82)**
*Whatsapp *(*n* = 825)		
Generation Z	**6.78 (3.67–12.52)**	**4.60 (2.52–8.39)**
Millenials	**4.62 (2.63–8.12)**	**3.72 (2.12–6.51)**
Generation X	**2.72 (1.61–4.60)**	**1.99 (1.18–3.37)**

Notes: Regression analyses were adjusted for variables such as gender, educational level and years with respiratory disease. Bolded values are significant at .05 significance level. ICTs, information and communication technologies; SMS, short message service; OR, odds ratio; CI, confidence interval. ^a^Reference generational cohort category is silent generation.

## Data Availability

The datasets used and/or analyzed during the current study are available from the corresponding author on reasonable request.

## Abbreviations

ICTs: Information and communication technologies
OLD: Obstructive lung diseases
OR: Odds ratio,
COPD: Chronic obstructive pulmonary disease
SMS: Short message service
SD: standard deviation.

## Supplementary Materials

Supplementary Material Supplemental Appendix: Table S1: frequency of use, to obtain information, and interest in receiving and asking for information through ICTs by generational cohort.
