# Supplementary material for: Influence of Generational Cohorts on the Preferences for Information and Communication Technologies in Latin American Patients with Obstructive Lung Diseases
Source: Int J Telemed Appl. 2020 Jan 23;2020:2489890. doi: 10.1155/2020/2489890 (PMC7212335; doi:10.1155/2020/2489890)
Supplement: Supplementary Material — Supplemental Appendix: Table S1: frequency of use, to obtain information, and interest in receiving and asking for information through ICTs by generational cohort. [file 2489890.f1.docx]

**Supplemental Appendix**

| **Table S1** – Frequency of use, to obtain information, and interest in receiving and asking for information through ICTs by generational cohort. | | | | | | | | |
| --- | --- | --- | --- | --- | --- | --- | --- | --- |
|  | Generation Z (n=114) | Millennials (n=163) | Generation X (n=166) | Baby Boomers (n=325) | Silent Generation (n=195) | G.I. Generation (n=5) | p-value | Total (n=968) |
| Internet Access | 95.1% | 93.7% | 77.3% | 49.5% | 36.2% | 0.0% | .000 | 63.6% |
| **Owning** | | | | | | | | |
| Cellphone | 95.2% | 96.3% | 95.0% | 86.1% | 70.6% | 0.0% | .000^a^ | 86.6% |
| Smartphone | 71.4% | 77.5% | 58.4% | 38.3% | 28.2% | 0.0% | .000 | 51.6% |
| **Use of ICT type (at least once a week)** | | | | | | | | |
| SMS | 63.5% | 76.8% | 85.6% | 56.9% | 40.5% | 0.0% | .000 | 63.2% |
| Facebook | 63.5% | 70.4% | 54.6% | 30.9% | 23.5% | 0.0% | .000 | 44.6% |
| Twitter | 31.8% | 33.6% | 13.4% | 5.7% | 6.1% | 0.0% | .000 | 15.4% |
| YouTube | 51.6% | 55.6% | 30.3% | 9.7% | 10.7% | 0.0% | .000 | 26.5% |
| Email | 51.5% | 67.3% | 51.3% | 29.8% | 28.0% | 0.0% | .000 | 42.5% |
| Internet | 68.8% | 72.5% | 60.1% | 39.2% | 31.2% | 0.0% | .000 | 50.5% |
| LinkedIn | 15.5% | 30.2% | 13.7% | 0.9% | 1.6% | 0.0% | .000 | 10.1% |
| Skype | 24.1% | 34.2% | 16.2% | 3.1% | 4.6% | 0.0% | .000 | 13.7% |
| **Uses ICT to obtain information about disease** | | | | | | | | |
| Internet | 52.8% | 55.2% | 46.5% | 27.5% | 17.3% | 0.0% | .000 | 36.9% |
| Facebook | 15.6% | 11.5% | 9.3% | 5.9% | 4.5% | 0.0% | .009 | 8.2% |
| Twitter | 2.3% | 3.7% | 3.6% | 1.7% | 0.7% | 0.0% | .297^a^ | 2.2% |
| YouTube | 10.1% | 14.4% | 9.0% | 3.5% | 2.6% | 0.0% | .000 | 6.9% |
| Email | 15.8% | 13.6% | 17.6% | 7.9% | 8.3% | 100.0% | .001^a^ | 11.6% |
| **Interest in receiving information through electronic media type (high/some interest)** | | | | | | | | |
| SMS | 51.5% | 56.2% | 62.3% | 55.6% | 45.1% | 0.0% | .039 | 54.4% |
| Facebook | 43.8% | 46.0% | 32.1% | 20.5% | 12.9% | 0.0% | .000 | 28.4% |
| Twitter | 31.0% | 20.1% | 6.7% | 1.5% | 1.4% | 0.0% | .000 | 8.8% |
| LinkedIn | 19.7% | 12.4% | 3.5% | 0.4% | 0.0% | 0.0% | .000^a^ | 5.1% |
| Email | 44.2% | 57.0% | 50.7% | 29.1% | 27.5% | 0.0% | .000 | 39.3% |
| **Interest in asking physician through ICT type (high/some interest)** | | | | | | | | |
| SMS | 54.0% | 59.5% | 68.9% | 72.0% | 77.0% | 100.0% | .000^a^ | 68.5% |
| Facebook | 45.3% | 40.1% | 28.3% | 30.9% | 22.9% | 0.0% | .001 | 32.1% |
| Twitter | 19.5% | 18.5% | 3.7% | 6.9% | 7.4% | 0.0% | .000 | 9.7% |
| LinkedIn | 16.4% | 14.9% | 1.8% | 2.2% | 1.5% | 0.0% | .000^a^ | 5.5% |
| Email | 42.6% | 53.8% | 47.0% | 16.9% | 11.7% | 0.0% | .000 | 31.1% |
| **Interest in receiving information through WhatsApp (Yes/No)** | | | | | | | | |
| Interested | 73.3% | 75.3% | 62.1% | 39.8% | 28.3% | 0.0% | .000 | 51.8% |
| **Interest in asking physician about disease through WhatsApp (Yes/No)** | | | | | | | | |
| Interested | 71.7% | 73.6% | 56.5% | 40.9% | 33.1% | 0.0% | .000 | 52.0% |
| Notes: All data are presented as percentages. Differences in values between the three age groups are significant at .05 significance level ^a.^ Fisher exact test performed | | | | | | | | |
